# Epidemiological link of a major cholera outbreak in Greater Accra region of Ghana, 2014

**DOI:** 10.1186/s12889-017-4803-9

**Published:** 2017-10-11

**Authors:** Kennedy Ohene-Adjei, Ernest Kenu, Delia Akosua Bandoh, Prince Nii Ossah Addo, Charles Lwanga Noora, Priscillia Nortey, Edwin Andrew Afari

**Affiliations:** Department of Epidemiology and Disease Control, School of Public Health, University of Ghana, Accra, Ghana

**Keywords:** Cholera, Epidemiological link, Greater Accra Region

## Abstract

**Background:**

Cholera remains an important public health challenge globally. Several pandemics have occurred in different parts of the world and have been epidemiologically linked by different researchers to illustrate how the cases were spread and how they were related to index cases. Even though the risk factors associated with the 2014 cholera outbreak were investigated extensively, the link between index cases and the source of infection was not investigated to help break the transmission process. This study sought to show how the index cases from various districts of the Greater Accra Region may have been linked.

**Methods:**

We carried out a descriptive cross sectional study to investigate the epidemiological link of the 2014 cholera outbreak in the Greater Accra region of Ghana. An extensive review of all district records on cholera cases in the Greater Accra region was carried out. Index cases were identified with the help of line lists. Univariate analyses were expressed as frequency distributions, percentages, mean ± Standard Deviation, and rates (attack rates, case-fatality rates etc.) as appropriate. Maps were drawn using Arc GIS and Epi info software to describe the pattern of transmission.

**Results:**

Up to 20,199 cholera cases were recorded. Sixty percent of the cases were between 20 and 40 years and about 58% (11,694) of the total cases were males. Almost 50% of the cases occurred in the Accra Metro district. Two-thirds of the index cases ate food prepared outside their home and had visited the Accra Metropolis.

**Conclusions:**

The 2014 cholera outbreak can be described as a propagated source outbreak linked to the Accra Metropolis. The link between index cases and the source of infection, if investigated earlier could have helped break the transmission process. Such investigations also inform decision-making about the appropriate interventions to be instituted to prevent subsequent outbreaks.

**Electronic supplementary material:**

The online version of this article (10.1186/s12889-017-4803-9) contains supplementary material, which is available to authorized users.

## Background

Cholera is a disease which affects the intestines and is characterized by severe watery diarrhea with vomiting and severe dehydration [1, 2]. About 75% of people infected with *Vibrio cholera* do not develop any symptoms, although the bacteria are present in their faeces for 7–14 days after infection and are shed back into the environment, potentially infecting other people. The mode of transmission of Cholera is through fecal contamination of food or water. Transmission is therefore closely associated to poor environmental management due water and sanitation issues [3, 4]. This makes the disease a key indicator of lack of social development [3]. Cholera is an extremely virulent disease that can kill within hours and affects both the young and old [5]. Its severity of diarrhea linked with vomiting can lead to rapid dehydration and electrolyte imbalance, which can eventually lead to death. About 50% of cholera cases may die if left untreated [3].

Globally, cholera remains an important public health challenge. It is estimated that 1.4 to 4.3 million cholera cases, and 28,000 to 142,000 cholera deaths occur globally each year [3, 4]. The global burden of cholera is large, particularly in Africa and southern Asia. Ghana recorded over 9000 cholera cases in 1999 with about 250 deaths [6]. In an outbreak from September 2010 to April 2011, there were over 8000 cases with 89 deaths from three regions of the country; Central, Eastern and Greater Accra [7].

Several studies have shown that poor environmental conditions, absence or shortage of safe water, as well as poor waste management are the main factors that promote cholera epidemics [6, 8–12].

Epidemiological links depict the importance of early investigations and how they can be used to ameliorate disease outbreaks. They also inform decision-making about the appropriate interventions to be instituted to prevent subsequent outbreaks. Several pandemics in different parts of the world and have been epidemiologically linked by different researchers to illustrate how the cases are spread and how they are related to index cases. Results from such studies have informed decision-making and effective interventions. This was initiated by the work of John Snow in 1854 on Cholera when his findings informed fundamental changes in water and waste systems in London [13].

Instituting interventions for the control of cholera mainly result from knowing the risk factors and the link between the index cases and this can curtail and prevent future outbreaks. Ghana recorded its worst outbreak of cholera in 2014 with eight out of its 10 regions being affected. The cholera outbreak started in June 2014 and continued to 2015. By the end of January 2015 when the outbreak was finally contained, over 28, 000 cases with 243 deaths had been recorded in all 10 regions of Ghana [14]. The Greater Accra Region (GAR) was the most affected, recording almost 98% of all cholera cases [14]. Though investigations were done intensively to identify the risk factors [10] as is done for almost every cholera outbreak in Ghana, no systematic effort have been put in place to measure the link that may exist between the index cases. There is the need to investigate the link between index cases and the source of infection to help break the transmission process.. This study therefore sought to show how the index cases from the various districts of the Greater Accra region may have been linked.

## Methods

### Study design and study area

**Fig. 1:**
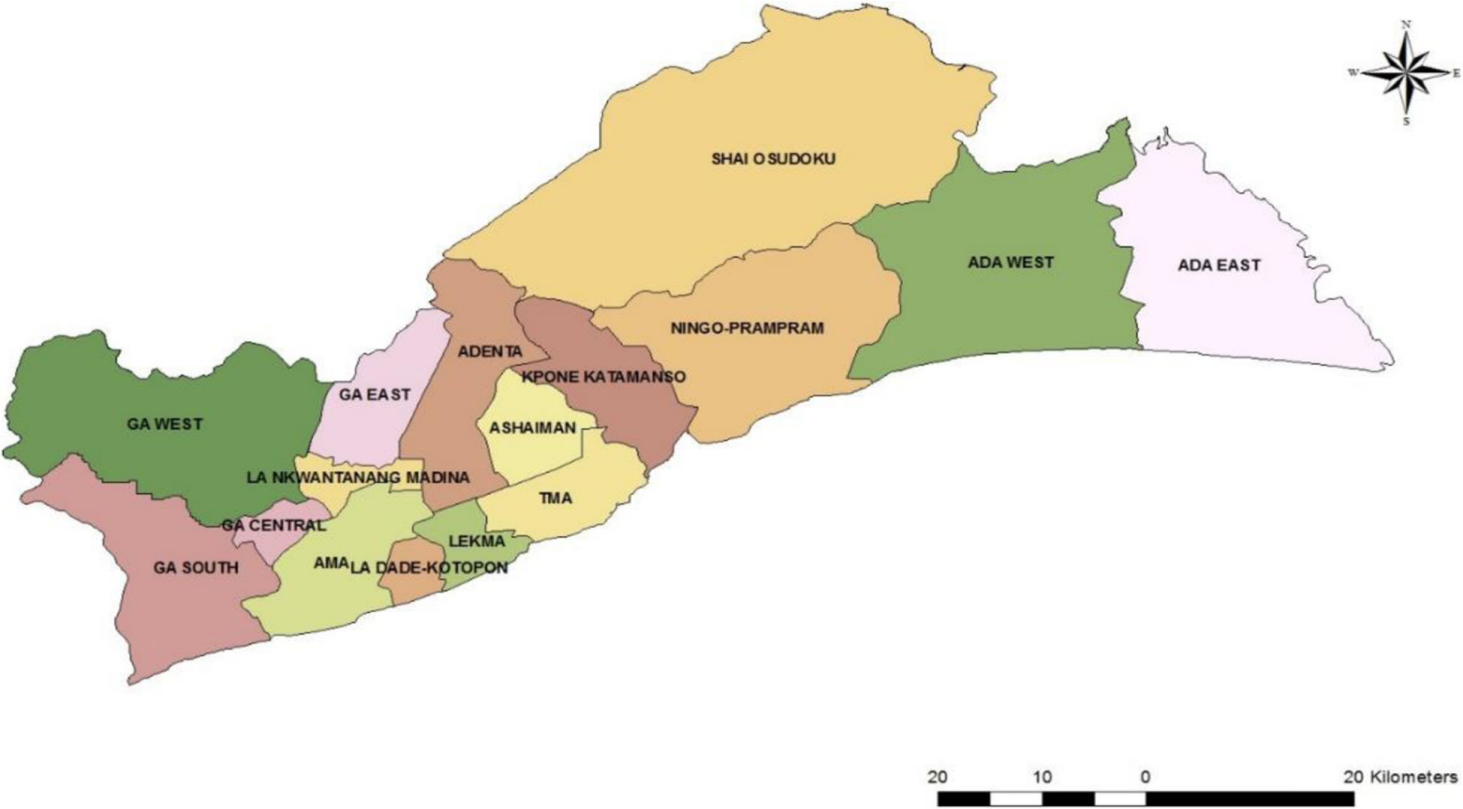
Map of Greater Accra Region showing its sixteen Districts

We carried out a descriptive cross sectional study to investigate the epidemiological link of the 2014 cholera outbreak in the Greater Accra region of Ghana (GAR). The Greater Accra Region has 16 local government administrative districts (Fig. 1). It lies in the South East of the country along the Gulf of Guinea and has miles of beautiful coastline especially in the rural parts of the region. It is the smallest of the 10 Administrative Regions of Ghana in terms of area, occupying 1.4% of the total land area of Ghana. However, the region contains 15.4% of Ghana’s population, making it the second highest populated region after Ashanti region. It has a growth rate of 2.8%, which is above the national average of 2.4% and has the highest population density in the nation. The 2014 Family Health Annual Report of the Ghana Health Service gave the population of Greater Accra Region as 4,530,905 [14]. The main source of drinking water for about 80% of the households in the region is pipe borne. However, the common source is outside the house. About 50% of the households in the region use mainly public toilet facility or water closets in their houses. Over half of the households in the region dispose of solid waste at public dumps.

### Study population

The study population consisted of all the index cases, both males and females regardless of their demographic characteristics in all districts, municipals and metropolitans that experienced the 2014 cholera outbreak in GAR. Index case was defined as the first documented case of a contagious disease in a group or population that serves to call attention to the presence of the disease.

There was an extensive review of records of the 2014 cholera outbreak in all the districts with cases by the use of the line lists. Affected districts were identified and visited. In the districts, line lists from facilities with cases were reviewed to identify their index cases, this was done with the help of the District Disease Control Officer. Identifying characteristics of the index cases were; the date of onset, date seen at health facility, name, client’s folder number and address. The folder number was then used to trace the client’s folder additional information such as contact persons and detailed residential address with telephone numbers. The Index case for each affected district was identified and traced by their details provided into their respective communities in GAR. They were then interviewed in their homes after consent had been obtained.

### Sources of data

Data for this study was obtained from both primary and secondary sources. Primary data included information from interview of index cases, environmental assessment and GPS coordinates. Secondary data was extracted from records obtained by reviewing existing line lists and client folders at the various levels from the various facilities.

### Data collection tools and techniques

An extensive review of all district records on cholera cases in the GAR was carried out. Index cases were identified with the help of line lists and cases’ folders used to obtain additional relevant information to trace index cases. A questionnaire was administered to all index cases in the affected districts to identify the possible modes by which they contracted the disease as well as their link to any of the cases from the other districts.

The linkage was drawn based on the history of visit, people contacted, as well as food and water consumed prior to the onset of the disease. An environmental assessment of the permanent residence of each index case was carried out and a digital camera was used to capture a photograph of the environment. The GPS co-ordinates of each residence was also taken by standing in front of the house.

### Data management and analysis

Data was cleaned, checked for consistency and analyzed using STATA version 13 and Arc GIS version 10. We performed descriptive analysis of the outbreak data by person, place and time. Univariate analyses were expressed as frequency distributions, percentages, mean ± SD, and rates (attack rates, case-fatality rates etc.) as appropriate.

Maps were drawn using Arc GIS to describe the pattern of transmission and show the areas where index cases live and the distribution of all cases by district. Different geographic areas (districts and communities) were shaded in different colours for easy identification and comparisons. Epidemic curves were also drawn to show patterns of 2014 cholera outbreak distribution for the region and the respective districts. Linkage was drawn based on the history of visit, people contacted and food and water consumed. Pictures of the environment of index cases taken were also presented as environmental analysis.

### Ethical consideration

Ethical approval was granted by the Ethical Review Committee of the Ghana Health Service. Formal permission was obtained from the Greater Accra regional disease control unit as well as at the district health administrations. In addition, the objectives of the study were explained to the participants (index cases) and their consent obtained through a written informed consent document before soliciting information on a scheduled date. Subject codes were used to ensure respondents’ identity remained anonymous and ensure confidentiality.

## Results

### Descriptive characteristics of cholera cases

Table 1 shows the demographic characteristics of cases recorded in the 2014 cholera outbreak in the Greater Accra Region. Up to 20,199 cholera cases were recorded. Children under 5 years of age recorded the least number of cases (2.8%). The mean age of the cholera cases was (29 ± 6.9) years. Sixty percent of the cases were between 20 and 40 years and about 58% (11,694) of the total cases were males.

**Table 1:** Descriptive characteristics of Cholera cases

Characteristics	Cases	Percent	Died(%CFR)	Chi2	*P*-value
Age Group				42.26	< 0.001^a^
Under 5 yrs	582	2.88	0		
6–19 yrs	4023	19.92	12(0.3)		
20–40 yrs	12,255	60.67	64(0.5)		
40 + yrs	3339	16.53	45(1.4)		
Sex				6.64	0.01^a^
Male	11,694	57.89	84(0.7)		
Female	8505	42.11	37(0.4)		

^a^Fisher’s exact test

*CFR* Case Fatality Rate

### Association between age, sex and case fatality rate (CFR)

There was an increase in CFR as age increases. Cases under 5 years recorded the least CFR (0) whilst cases above 40 years recorded the highest CFR (1.4%). CFR was also higher in males (0.7%) than in females (0.4%) (Table 1).

### Distribution of cases by person, place and time

Cholera cases were reported in 15 out of the 16 districts. The Accra Metropolis recorded the highest number of cholera cases (52%, 10,504). The least number of cases were seen in Ningo Prampram district (0.11%, 22). Ashaiman and Ningo Prampram did not record cases below the age of 5. (Table 2).

**Table 2:** Age group distribution of cholera cases by districts

District	Age Group				Total / (%)
	< 5 yrs	6 – 19 yrs	20 – 40 yrs	40 + yrs	
Accra Metro	210	2206	6512	1576	10,504/ (52)
Ada East	1	18	56	28	103/ (0.51)
Adentan	5	10	29	5	49/ (0.24)
Ashaiman	0	10	49	9	68/ (0.34)
Ga Central	11	35	76	31	153/ (0.76)
Ga East	7	42	115	26	190/ (0.94)
Ga South	146	423	1089	297	1955/ (9.68)
Ga West	22	264	723	328	1337/ (6.62)
Kpone Katamanso	23	62	214	70	369/ (1.83)
La Dadekotopon	28	335	1747	217	2327/ (11.52)
La Nkwatanang	10	52	210	54	326/ (1.61)
Ledzoku Krowor	12	256	717	462	1447/ (7.16)
Ningo Prampram	0	2	14	6	22/ (0.11)
Shai Osu Doku	39	64	109	34	246/ (1.22)
Tema	68	244	595	196	1103/ (5.46)
**Greater Accra**	**582**	**4023**	**12,255**	**3339**	**20,199/(100)**

**Fig. 2:**
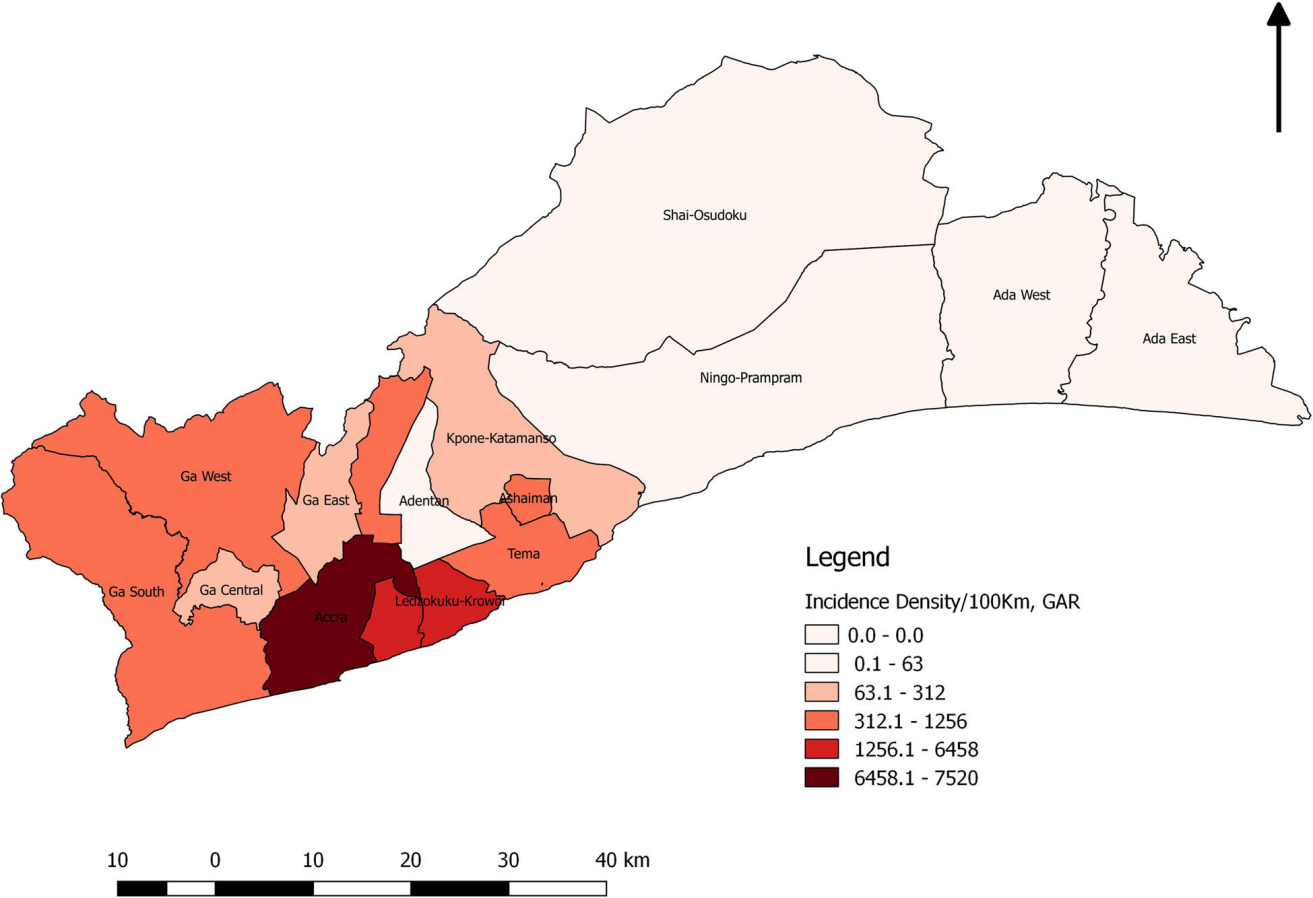
Spot map of Cholera cases in the Greater Accra Region during the 2014 outbreak

Ada West district did not report any cases. The districts with high case fatality rates were La Nkwantanang, Ashiaman, Ledzokuku Krowor, Ada East and Tema metro (Refer to Additional file 1). The region recorded an attack rate of 445 per 100,000 population with Case Fatality Rate (CFR) of 0.60% (Table 3). Fig. 2 shows a spot map of cholera cases by districts and shows that a higher burden of cases were found in the Western part of the Region.

**Table 3:** Attack rates and case fatality rate of cholera by districts

DISTRICT	2014 POP.	TOTAL CASES	DEATHS	AR/100,000 POP	%CFR
Accra Metro	1,857,558	10,504 (0.57%)	65	565.47	0.62
Ada East	83,515	103 (0.12%)	1	123.33	0.97
Adentan	88,374	49 (0.06%)	0	55.45	0.00
Ashaiman	215,777	68 (0.03%)	1	31.51	1.47
Ga Central	116,926	153 (0.13%)	1	130.85	0.65
Ga East	165,274	190 (0.11%)	0	114.96	0.00
Ga South	431,795	1955 (0.45%)	5	452.76	0.26
Ga West	296,868	1337 (0.45%)	2	450.37	0.15
Kpone Katamanso	109,184	369 (0.33%)	0	337.96	0.00
La Dadekotopon	231,166	2327 (1.00%)	15	106.64	0.64
La Nkwatanang	128,120	326 (0.1%)	6	254.45	1.84
Ledzoku Krowor	257,538	1447 (0.25%)	14	561.86	0.97
Ningo Prampram	78,006	22 (0.03%)	0	28.20	0.00
Shai Osu Doku	60,785	246 (0.40%)	1	404.71	0.41
Tema	345,750	1103 (0.32%)	10	319.02	0.91
**Greater Accra**	**4,530,905**	**20,199 (0.45%)**	**121**	**445.80**	**0.60**

**Fig. 3:**
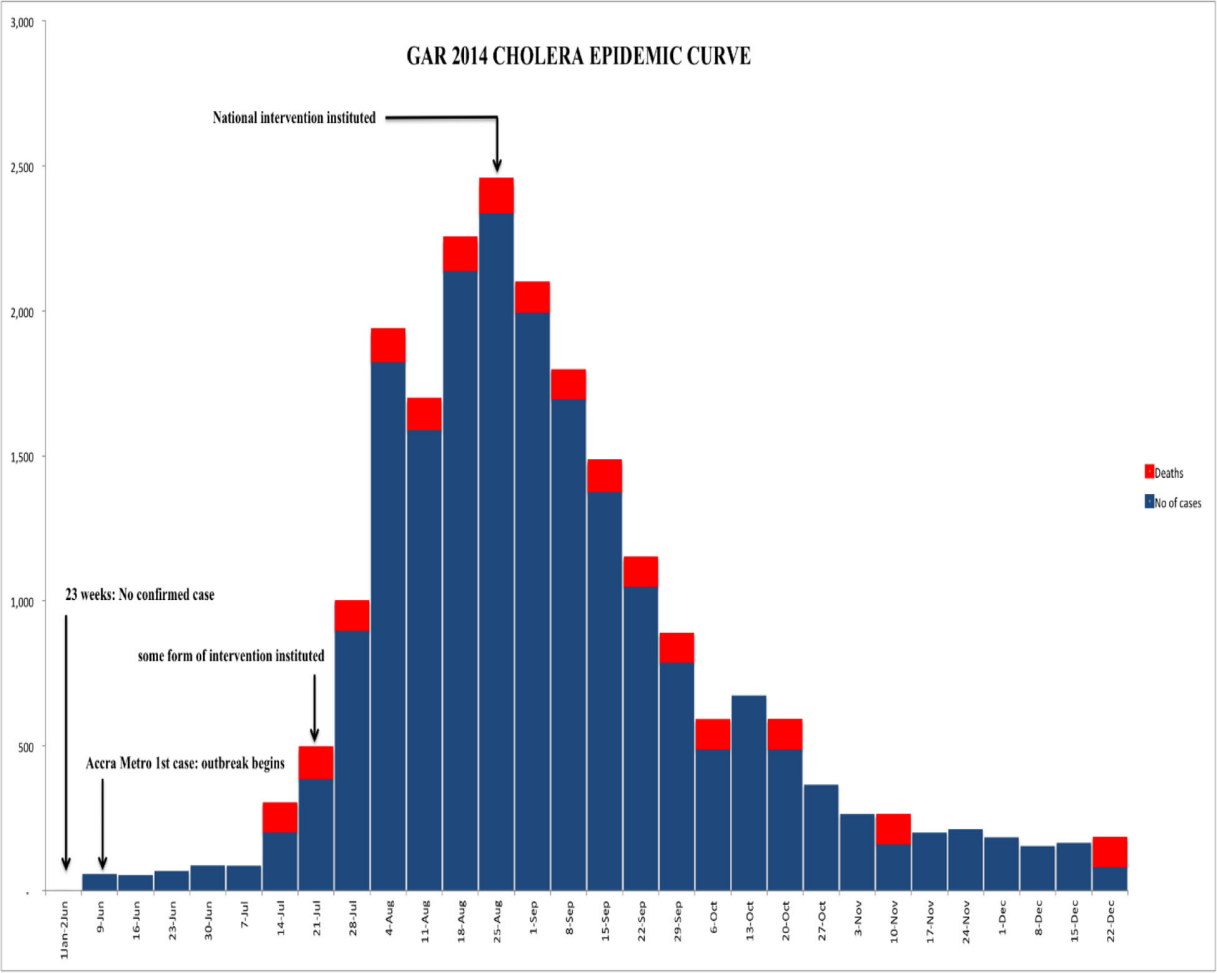
Cholera epidemic curve

The epidemic curve describes the 2014 cholera outbreak as a propagated outbreak. The year started with no confirmed cases recorded for the first 23 weeks in the GAR. The first positive case was confirmed in week 24 (9th June 2014), when the epidemic began. Five more districts confirmed between week 27 (30th June, 2014) and 29 (14th July, 2014). By the 33rd week (11th August, 2014), all districts in the region had reported cholera cases with the exception of Ada West. The epidemic reached its peak during the 35th week (25th August, 2014) and drastically reduced when national interventions to control the outbreak were initiated. It then declined steadily during the 49th week (1st December, 2014). Few cases were still reported till the end of the year but the pace of reporting was slower (Fig. 3).

Epidemic curves for the affected districts can be found in the Additional file 1.

### Link between index cases and various districts

A total of 15 index cases were identified and interviewed from the 15 affected districts. GAR 2014 cholera outbreak started when a 10-year-old girl reported to the Ussher Polyclinic, a facility in the Accra Metropolis. She reported on 10th June, 2014 with onset of the disease from the previous day. Stool specimen from her proved positive to Vibrio cholerae 01 of the Ogawa sub type. The laboratory confirmation came in after she had been discharged. Follow up investigation revealed that the evening prior to the onset of diarrhoea she ate rice and fried turkey tail (locally called Tsofi) from a street food vendor at Agbado, a suburb of Accra Metropolis. Another case was confirmed in Maamobi polyclinic, another facility in the Accra Metropolis within the same week. After the second confirmed case, the Accra Metro began to experience an upsurge in the number of cholera cases reporting. The next affected district after Accra metropolis was Adantan municipal whose first case reported on 1st July 2014. This was a woman who trades at the Makola market, believed to have gotten the infection whiles there and exported the disease to her district. Ashiaman recorded their first case on 4th July 2014. The case was a 24 yrs. old male who came on vacation from school and usually visits his friends who runs an internet café in Accra. He spends most of his with them and eats from there. All the other districts had a contact in the Accra metropolis with the exception of Tema and Kpone Katamanso. Kpone Katamanso district index case can be traced to Tema where a caterer visits Tema on commercial purposes during which there was an ongoing cholera transmission. She gets herself infected and exports the infection to her community, Apolo in Kpone. Tema’s index case was a fisherman who is always on the sea fishing. He started experiencing signs and symptoms of the disease on 19th July 2014 when by then there were existing outbreaks in six districts. Tema is surrounded by four out of the six districts, ie. Adentan, Ashiaman, La Dadekotopon and Ledzokuku Krowor. However, Tema and Accra metro are linked to each other by the sea were fishing activities take place.

Results from the interview reveals that 80% (20) had traveled or visited the Accra metropolitan areas. There were various communities in the Accra Metro where index cases visited, these places included; Darkuman, Dansoman, Chorkor (Ablekuma sub-metro), Lapaz, Achimota (Okaikoi sub-metro), Kwame Nkrumah circle (Osu Clottey sub-metro) and Accra central (Ashiedu Keteke sub-metro). Two-thirds of the index cases ate food prepared outside their home and had visited the Accra Metropolis (Table 4).

**Table 4:** Link between index cases of the 2014 cholera cases in GAR

Sequence of index cases	Date of onset	District reporting index case	Community index case lived	Places visited prior to symptoms	District (visited visited)	Source of food/water
1	9/6/14	Accra Metro	Agbado	did not visit anyone	Accra Metro	Bought food across the street
2	1/7/14	Adentan	East legon	Mother is a trader, buys food stuff from Makola	Accra Metro	Bought food for the child at Makola
3	4/7/14	Ashiaman	Klagon	Visited some friends in Accra during his vacation	Accra Metro	ate in Accra during his visit
4	7/7/14	Ga West	Tentra Hill	Schools at Achimota	Accra Metro	Bought food from school
5	9/7/14	La Dade Kotopon	Weija	visted the auntie in Dansoman	Accra Metro	could not remember
6	14/7/14	Ledzokuku Krowor	Teshie Salem	didn’t go anywhere apart from school	LedzokukuKrowor	Bought food from outside
7	19/7/14	Tema Metro	Awudun	A fisherman, goes to the sea all the time	Tema Metro	could not remember
8	21/7/14	Ga East	Ashongman	Sells at Kwame Nkrumah circle	Accra Metro	Bought food from circle
9	23/7/14	La Nkwantanan	Agbogba	Mother didn’t know where he went to	Ga East	mother could not tell
10	23/7/14	Ga Central	Santa Maria	Child attends school in Lapaz	Accra Metro	Bought food from school
11	23/7/14	Ga South	Anyaa	Trader at Lapaz	Accra Metro	Ate and drinks in Lapaz
12	30/7/14	Shai Osu Doku	Dodowa	Went to Accra to buy something a day before onset	Accra Metro	Ate in Accra
13	3/8/14	Kpone Katamanso	KponeApolo	A caterer, goes to tema to buy food stuff	Tema Metro	Could not remember
14	5/8/14	Ada East	Korlekope	came from Chorkor to visit a relative on Aug 4	Accra Metro	Ate home prepared food
15	12/8/14	Ningo Prampram	Darkuman	Lives in Darkuman, had his service in Ningo Prampram	Accra Metro	Patronize food vendors

### Environmental assessment

**Fig. 4:**
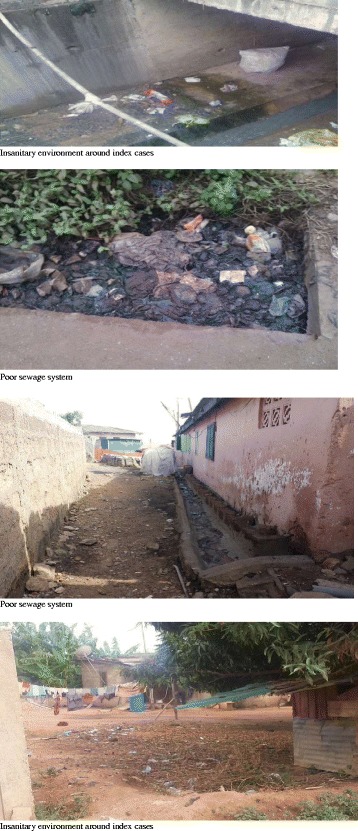
Example of environmental condition where district index cases live

Environmental conditions of the communities within which index cases for the various districts lived were assessed. Pictures (Fig. 4) of the environment were taken to be able to well describe it. The various environmental conditions within which index cases were found can be described as unhygienic and unclean. There was generally poor environment sanitation. There was crude dumping in most of the places visited. There was open defecation in some of the places. This was because most of the households did not have a place of convenience attached to them. Inhabitants patronize the public latrines. The cesspit emptier that empties these liquid wastes end up emptying its content into the sea. There were inadequate drainage and sewage systems. Inhabitants resort to dumping at sea and in gutters when it rains. Continuous water supply was another major problem in these areas. They mostly depend on the sachet water as the safest source of drinking water.

## Discussion

This study determined the epidemiological links between the index cases in the 15 districts which were affected by the 2014 cholera outbreak in Accra metropolis. The outbreak eventually spread across 15 of the 16 districts in GAR and beyond. The region recorded more than 20,000 cases with 121 deaths with an attack rate of 445 per 100,000 population and a Case Fatality Rate (CFR) of 0.6%. Even though cholera has become endemic in the capital city particularly in the last decade, the 2014 outbreak recorded the highest attack rates and spread in recent history. This stands to reason that, the effects of a growing city in a developing country particularly the influx of population amidst challenges of waste management and disposal visa vi the activities of the population could have contributed to the wide spread and high numbers recoded in 2014 cholera outbreak.

In this study, more than half (52%) of the total cases came from the Accra Metropolis. Accra Metropolis recorded about four times the number of cholera cases the second most affected district recorded. Eighty percent of the index cases interviewed had visited the Accra metropolitan area. Similar preponderance of cholera cases in Accra Metropolis was observed by Gershon et al. in 2014 [8]. This could probably be due to the high population density in the Accra metro. Accra metro is the most urbanized and highly commercialized district in the region. There is high daily influx of traders, travelers, private personnel and civil workers from neighboring districts and other regions all over Ghana. Such high commercial activity results in overcrowding, straining of existing sanitation and social amenities, thereby putting people at an increased risk of cholera transmission. Two earlier studies done in Ghana also reported that the highest cholera cases were recorded in Accra [6, 8].

Environmental assessment of the residence of index cases indicated inadequate water supply, poor sanitation and unsafe disposal of solid and liquid waste. Also, since there is usually inadequate and inconsistent water supply in the community, people normally fetch water in buckets and gallons and store them for future use. These gets contaminated during the process of handling. Poor environmental sanitation could lead to contamination of food and thus might be the likely reason why 73% (11 of 15) the index cases who took water or food from venders outside their homes might have gotten infected and the also spread the disease in their communities.

This current finding confirms studies conducted by Emch in 2008 in a cholera outbreak in Bangladesh and another by Schaetti et al. on Tanzania’s cholera outbreak in 2009, where their identified risk factors included inadequate water supply, poor sanitation and unsafe disposal of solid and liquid waste [9, 10].

This study utilizes the advancement of the Geographic Information System especially with spatial disease mapping where the index cases were located and shows how the disease travelled within districts in the Greater Accra region. Visual observation of the map reveals heavy cholera transmission in the Accra Metropolis (Fig. 2), which forms the central part of the region. The transmission at the other districts can be attributed to the transmission occurring at the metropolis.

Based on interviews from the index cases, the 2014 cholera outbreak in GAR could be described as a propagated source epidemic, linked to Accra Metropolis (a 10-year-old girl from Agbado). Kpone Katamanso district is believed to have acquired the cholera transmission from Tema. Cholera transmission in Tema began on 19th July 2014 by which there were ongoing transmission in Accra metro, Adentan, Ashiaman, Ga west, La dadekotopon and Ledzokuku Krowor districts. Since Tema’s index case was a fisherman, there is a high probability that his infection was from Accra Metro than the other districts since Accra metro also have fishing communities. Seawater pollution is seen to be worse in the metropolis where the commonest contamination is from human excreta and sewage. Defecating and dumping in and at the banks of the sea and has become a common practice in the metropolis and unfortunately some urban inhibitors resort to such polluted water sources for various household activities for cooking and washing during periods of water shortage.

At the peak of the outbreak, all stakeholders including the district assemblies, Ghana water company, local government, ministry of health and Ghana Health Service, etc. were brought on board to help tackle the outbreak based on research which had been done on the outbreak. Interventions such as community education and sensitization on cholera, closing of wells, environmental cleaning through community labour, provision of safe water and appropriate toilet facilities were put in place. The magnitude of outbreak steadily reduced after 6 weeks of intervention implementation.

This suggests that, if the control measures were instituted immediately after the first few cases were confirmed, the number of cases and deaths recorded could have been reduced considerably.

There were however some limitations to this study. Records on cases reviewed at the various level may just be a fraction of cases that actually occurred since there may have been some unreported cases in the communities, records at the various levels (regional, districts and facility) were therefore compared to fill in the missing pieces. There were difficulties in locating the homes of the index cases due to the poor addressing system in the region. There is a high tendency of recall bias among index cases since they were interviewed months after the outbreak had ended, the information recorded in their folders at the hospital was therefore taken along during the interview to help them remember some of events.

## Conclusion

The study has shown that the outbreak was a propagated source epidemic that can be linked to Accra metropolis. Two-thirds of the index cases ate food prepared outside their home. The study also affirms that urbanization and overcrowding resulting in insanitary conditions coupled with heavy rainfall and potable water shortage is a predictor of cholera outbreak in Ghana. It study provides a framework that can be used for future epidemiological research on infectious and environmentally related diseases.

## Additional file

Additional file 1: Copy of GAR_Cholera Linelist_latest, Epicurves for the districts. (ZIP 722 kb)

## Abbreviations

CFR: Case Fatality Rate
GAR: Greater Accra Region
GPS: Global Positioning System
