# Supplementary material for: Epidemiological link of a major cholera outbreak in Greater Accra region of Ghana, 2014
Source: BMC Public Health. 2017 Oct 11;17:801. doi: 10.1186/s12889-017-4803-9 (PMC5637323; doi:10.1186/s12889-017-4803-9)
Supplement: Additional file 1: — Copy of GAR_Cholera Linelist_latest, Epicurves for the districts. (ZIP 722 kb) [file 12889_2017_4803_MOESM1_ESM.zip › Add 1 Epicurves for the districtsR3.docx]

Epicurves fro the districts
